# Prevalence and risk factors associated with SARS-CoV-2 infections among veterinary practitioners and dogs patients, June-August 2020, Lagos, Nigeria

**DOI:** 10.1186/s42522-022-00062-1

**Published:** 2022-03-24

**Authors:** Folajimi O. Shorunke, Emmanuel C. Okolocha, Grace S. Kia, Aishat Usman, Oluseyi Akano, Emmanuel J. Awosanya

**Affiliations:** 1 Nigerian Field Epidemiology and Laboratory Training Program, Abuja, Nigeria; 2 African Field Epidemiology Network, Abuja, Nigeria; 3 Department of Veterinary Public Health, Faculty of Veterinary Medicine, Ahmadu Bello University, Zaria, Nigeria; 4 Department of Veterinary Public Health and Preventive Medicine, Faculty of Veterinary Medicine, University of Ibadan, Ibadan, Nigeria

**Keywords:** Veterinary Practitioners, Dogs, COVID-19, Zoonotic, Occupational risk, Lagos

## Abstract

**Background:**

Lagos State is the epicenter of COVID-19 in Nigeria, having the highest number of cases and death. Veterinary practitioners play an important role in public health and risk exposure to SARS-CoV-2, the virus that causes COVID-19 while attending to animal patients and owners. We determined the prevalence of covid-19 among veterinary practitioners and their dog patients in Lagos, and the associated risk factors during the lockdown.

**Methods:**

We administered questionnaires, reviewed clinical records and conducted serological test to determine the COVID-19 status of 112 veterinary practitioners and 154 dog patients and to assess the associated factors in nine veterinary clinics or hospitals between June to August 2020. Data were analyzed using descriptive statistics, bivariate and multivariate analyses at 5% significant level.

**Result:**

The mean age of participants was 37.5±10.5 and 66 (58.9%) were male. COVID-19 cases among the veterinary practitioners and dogs were 29 (25.9%) and 3 (2%), respectively. Predictors of COVID-19 cases among veterinary practitioners were contact with a SARS-CoV-2 infected dog (AOR: 25.42; CI 4.73-136.66); being a veterinary doctor working during the lockdown (AOR: 6.11; CI 1.56-24.00) and not disinfecting examination table after attending to dogs (AOR: 12.43; CI 1.39-110.99).

**Conclusion:**

Veterinary practitioners and dogs in Lagos were exposed to SARS-COV-2 virus during the COVID-19 lockdown. Contact with SARS-CoV-2 infected dogs, being a veterinary doctor working during the lockdown and not disinfecting examination tables after clinical examination were predictors of COVID-19 cases among veterinary practitioners in Lagos State. Strict Infection, Prevention and Control measures are recommended in veterinary practice.

**Supplementary Information:**

The online version contains supplementary material available at 10.1186/s42522-022-00062-1.

## Background

SARS-Coronavirus-2 (SARS-CoV-2) causes COVID-19 disease which was first reported in December 2019 in Wuhan China [1, 2]^.^ The virus was thought to have emerged from an animal source and spilled-over to human population. Although genetically closely related viruses were isolated from Rhinolophus bats [3], the source of SARS-CoV-2 and its route of introduction into human population is unknown. The first official case of COVID-19 in Nigeria was reported on February 27, 2020 [4] when a patient of Italian origin, arrived in Lagos from Europe. As at 9^th^ July 2021 there were 168,110 confirmed cases with 2,129 deaths (CFR 1.3%) across all the 36 states in Nigeria,[5] with Lagos having the highest cases 60,097 (36%) and death 456 (22%).

As part of the COVID-19 response in Nigeria, lockdown measures were instituted which restricted movement of persons and other activities. However, essential services such as hospitals, emergency services, security services, pharmacies, electricity and gas, food supply chains and veterinary establishments were in operation. [6, 7]

In Lagos State, veterinary practitioners work in both the public and private sectors delivering services such as animal disease diagnosis and treatment, vaccine administration, public health care, drug distribution, health education and providing care for animal patient. Some of the services rendered encourage close contact with animals and animal owners which may promote exposure to SARS-CoV-2 amidst limited supply of PPE which were in high demand in human hospitals [8].

Although the current pandemic of COVID-19 is being sustained through human to human transmission, animal infections with SARS-CoV-2 have however been reported in some countries. Several animal species have been reported to be infected with SARS-CoV-2 either naturally or experimentally.[9] Evidence from risk assessments, epidemiological investigations, and experimental studies do not suggest that live animals or animal products play a key role in SARS-CoV-2 infection in humans.[10] However, several animal species have tested positive for SARS-CoV-2, mostly as a result of close contact with humans infected with COVID-19 disease. [9] It is important to monitor infections in animals to better understand their epidemiological role in the maintenance and spread of SARS-CoV-2. With the increase in cases of COVID-19 infections and the occurrence of community transmission of the disease in various communities in Lagos Nigeria, and with the fact that veterinary practitioners were one of the essential workers in Lagos during lock down, [7, 8] we determined the prevalence of COVID-19 infection among veterinary practitioners, dogs patients presented at veterinary clinics; and factors associated with the infection. during the lockdown.

## Methods

### Study setting

The study was done in Lagos State, Nigeria. Lagos State is a port city located in the Southwestern geopolitical zone of Nigeria and has the smallest landmass of all the 36 states in the country measuring 3,577 sq km.[11] It is the most populated state in Nigeria with a population of 9,013,534 during the 2006 national census [12, 13] and a projected population of 14, 009,120 by 2020. Lagos state is divided into five administrative regions and 20 local government areas (LGA).[14]

Lagos was the epicenter of the outbreak of COVID-19 in Nigeria. There are currently 30 laboratories capable of testing for COVID-19 by RT-PCR in Lagos State: 5 public laboratories and 25 approved private laboratories.[15] There were eight isolation centers located in Lagos State during the lockdown period for the isolation and treatment of COVID-19 positive patients, with a 547 bed capacity.[15] A private hospital later received approval to manage clients willing to pay for their services.[15]

### Study design and study population

We conducted a cross-sectional study involving veterinary practitioners and canine patients presented in veterinary clinics in Lagos State.

#### Human

Inclusion criteria: All veterinary practitioners in selected private and public clinics or hospitals, working as frontline veterinary workers in Lagos during the period of the COVID-19 pandemic lockdown, from June to August 2020.

#### Animal

All dogs, attended to or boarded in selected private and public clinics or hospitals in Lagos during the period of the COVID-19 pandemic from June to August 2020.

Inclusion Criteria: All dogs that the owners gave permission to be sampled.

Exclusion Criteria: All dogs in the inclusion criteria that cannot be restrained for sample collection.

### Sample size determination

Sample size of veterinary practitioners:

$n=\frac{{Z}_{1-a/2}^{2}\;p(1-p)}{d}^{2}$ - As described by Omair [16]

Where: *n* = desired sample size

*Z* = value for the corresponding confidence level (e.g., 1.96 for 95% confidence)

*p* = estimated value for the proportion of a sample/expected prevalence (6%) [17]

*q* = 1-P,

*d* = is the margin of error (e.g., 0.05= ± 5%)

*n* =$$\frac{{1.96}^{2}\times 0.06(1-0.06)}{{0.05}^{2}}=87$$

A minimum sample size of 87 respondents was estimated.

Sample size of dogs tested.

All dogs in inclusion criteria (total sampling).

### Sampling technique

**Fig. 1:**
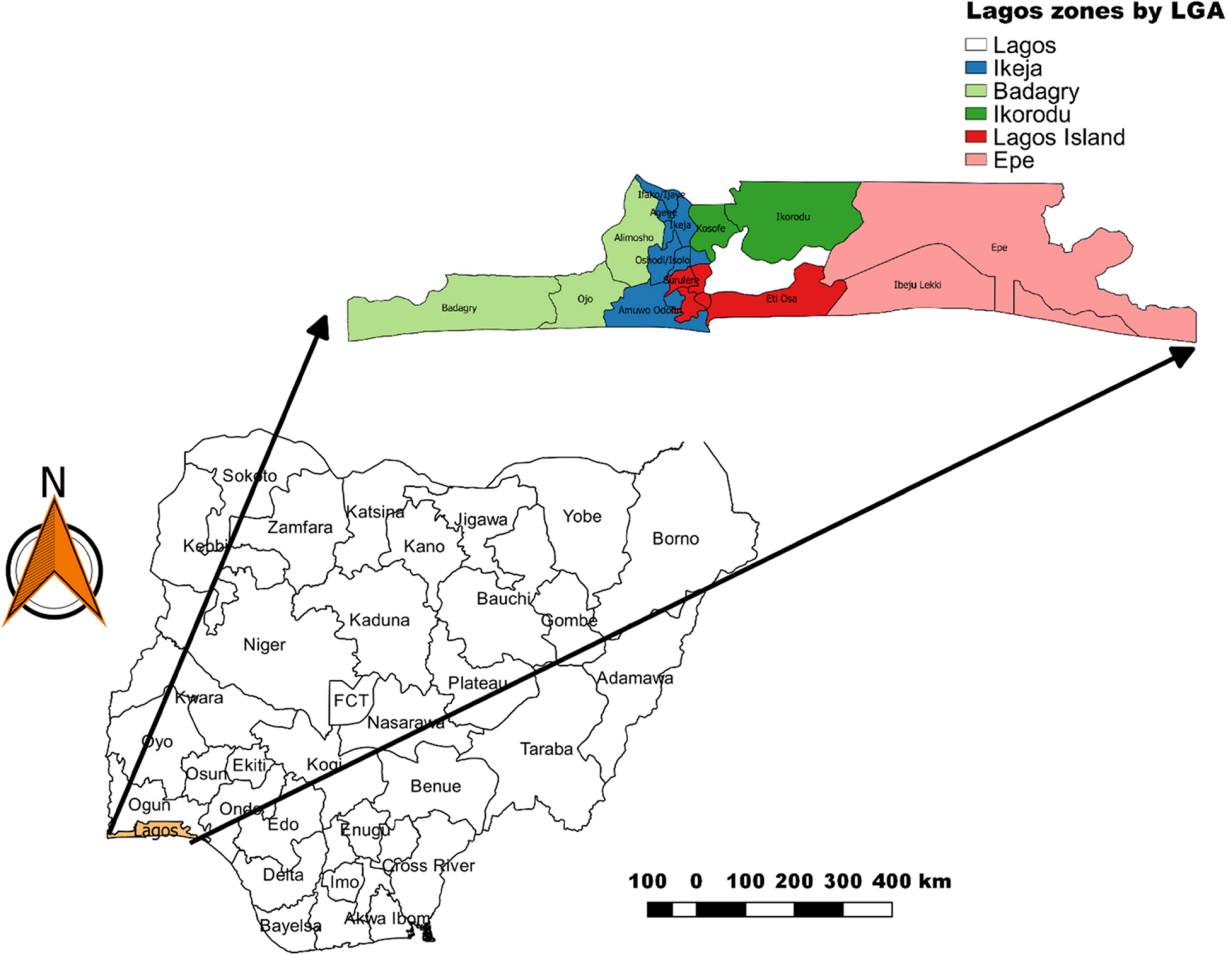
Map of Nigeria, with the zonal division of Lagos. This shows the map of Nigeria, highlighting the zonal division of Lagos State into five, with each zones representing a different color. The zonal division was used during sampling, to choose the appropriate veterinary clinic to be selected for sample collection for representativeness

A multistage sampling technique with 3 stages was adopted for the study. Stage 1: Lagos State is divided into five divisions based on the geographical and socioeconomic distribution of the state (fig. 1) which is also the distribution of the location of public veterinary health facilities in Lagos.

The five divisions are Ikeja, Badagry, Ikorodu, Lagos Island and Epe divisions. State public primary veterinary health facilities within these zones, making up to five primary veterinary health clinics were selected (complete sampling). In addition, four private veterinary clinics that operated during the lockdown were randomly selected from the same division as the public veterinary facility earlier selected this included Ikeja, Ikorodu, Lagos Island and Badagry divisions. Stage 2: total sampling of all the nine veterinary health facilities was done. Stage 3: All Veterinary practitioners that worked in selected clinics during the period of the lock down, from June to August 2020, were selected and a semi-structured questionnaire administered to them either electronically or by an interviewer.

### Case definition

Confirmed cases for human subject were determined using the case definition by the Nigeria Center for Disease Control (NCDC).[18] which defined a confirmed case as “Any person with laboratory confirmation of SARS-CoV-2 infection through PCR. Confirmed cases for animal subject was any dog with IgG/IgM antibody RDT detection with or without signs and symptoms.

### Active case search

In September 2020 we reviewed the hospital case file register to select the dogs attended to at the clinics during the study period. All the dogs that fit the inclusion criteria were selected for blood sampling after obtaining approval from the owners.

### Study instruments and data collection

Two questionnaires were developed for this study using the Kobotool: the first is for veterinary practitioners. We piloted this questionnaire by collecting data from four veterinarians not included in the study sample. Feedback on their understanding of each question was received and questions were adjusted accordingly. The average completion time of the questionnaire was 10 minutes. The final questionnaire had structured, closed-ended questions with responses based on multiple-choice. The veterinary practitioner questionnaire had two sections: the first section consisted 11 questions on socio-demographic variables such as age, sex, educational qualification, marital status, household number; management factors which included years of experience, work cadre, clinic types, clinic locations; and COVID-19 status; while the second section consisted of six questions assessing the level of contact with dogs by the veterinary practitioners and their adherence to COVID-19 IPC while on duty.

The questionnaire was administered to the veterinary practitioners by trained interviewer or electronically through the department of veterinary services WhatsApp group. The COVID-19 status of the respondents were then verified through Surveillance Outbreak Response Management and Analysis System (SORMAS). The second questionnaire was administered to the dog owner or handler and used to obtain dogs demographics, which included age, sex, LGA of residence, clinic presented at and dog breed at both public and selected private veterinary health facilities throughout Lagos.

### Sample collection method

All dogs attended to by the veterinary practitioners in the nine selected veterinary clinics were screened for IgG/IgM SARS-CoV-2 antibodies using Uni-science COVID-19 RDT test kit.[19] Blood samples (5ml) were collected into plain sample bottles, preserved on ice packs between 4^0^c to 8^0^c and transported to the laboratory for analysis. Sera were obtained from the clotted blood samples after centrifugation at 1500rpm for 10minutes. Serological test was carried out according to manufacturer’s instruction. Test results were communicated to the participating clinics for proper handling of positive cases.

### Data analysis

We conducted descriptive statistics to summarize the data in form of frequencies, proportions and percentages using Microsoft Excel (2016). Maps of distributions of positive cases among veterinary practitioners and dogs based on location was also obtained using QGIS. Bivariate analysis was conducted to determine factors associated with COVID-19 infection at *p*< 0.10. Multivariate logistic regression was used to identify independent predictors at *p*< 0.05 using Epi-info version 7.

### Ethical approval

Ethical approval for the animal component of the research was obtained from the Lagos State Veterinary Research Ethics Committee (Reference number: LAGVREC/PRS/569T/159). Which is a body that has the responsibility of granting approval for research done within the ministry of agriculture Lagos State. For the human component of the research, ethical approval was obtained from the Osun state Health Research Ethics Committee (OSHREC) to obtain data from veterinary practitioners in-terms of their COVID-19 exposures (Reference number OSHREC/PRS/634T/191).

## Results

A total of 112 veterinary practitioners working in six LGAs in Lagos State participated in the study with a 100% response rate. The mean age of participants was 37.5±10.5 and 66 (58.9%) were male. More than half, 76 (68%) resided in urban areas of the state with the highest 44 (36%) from Ikeja LGA, 29 (25.9%) of the respondents returned a positive COVID-19 result (Table 1).

**Table 1:** Socio-demographic profile of respondents in the study from June – August, 2020 (*n* = 112)

**Variables**	**Frequency**	**Percentage**
**Gender**		
Male	66	58.9
Female	46	41.1
**Age in years**		
20-29	34	30.3
30-39	28	25.0
40-49	32	28.6
50-59	16	14.3
≥60	2	1.8
**Highest education**		
Below tertiary education	28	16.0
Tertiary education	84	84.0
**Marital status**		
Single	36	32.1
Married	76	67.9
**Number of household members**		
<5	66	58.8
≥5	36	32.4
No response	10	8.8
**Cadre of staff**		
Veterinary doctor	60	53.6
Others	52	46.4
**Years of work experience**		
≤10	74	66.1
11-20	26	23.2
21-30	10	8.9
≥31	2	1.8
**Type of resident**		
Semi-urban	36	32.2
Urban	76	67.8

A total of 3 (2.0%) of 154 dogs tested returned a positive SARS-CoV-2 infection result, all below 12 months of age, and were brachycephalic breeds (Table 2). A total of 80 (51.9%) presented to Government clinics and the largest proportion 48 (31.2%) resident in Ikeja LGA.

**Table 2:** Demographic profile of dogs presented to veterinary practitioners during the study period from June – August, 2020 (*n* = 154)

**Variable**	**Frequency**	**Percentage**
**Sex**		
Male	77	50
Female	77	50
**Age (months)**		
≤12	45	29.2
13-60	83	53.9
>60	26	16.9
**Breed type**		
Brachycephalic	71	46.1
Non brachycephalic	83	53.9
**Presentation by Clinic**		
Clinic 1	18	11.7
Clinic 2	14	9.1
Clinic 3	40	26.0
Clinic 4	22	14.2
Clinic 5	8	5.2
Clinic 6	12	7.8
Clinic 7	10	6.5
Clinic 8	20	13.0
Clinic 9	10	6.5
**LGA of resident**		
Badagry	12	7.8
Eti-osa	10	6.5
Ibeju Lekki	10	6.5
Ikeja	48	31.2
Ikorodu	31	20.1
Surulere	20	13.0
**Type of clinic attended**		
Private	74	48.1
Government	80	51.9
**SARS-CoV-2 serological test results**		
Positive	3	2.0
Negative	151	98.0

The highest proportion of COVID-19 cases among veterinary practitioners was in clinic 3 which also had 5.0% of the dogs tested positive. None of the veterinary practitioners in clinic 2 was positive, however it had the highest proportion of positive cases among dogs (Table 3).

**Table 3:** COVID-19 cases among veterinary practitioners and dogs presented at clinics in Lagos, June – August 2020

Clinic	COVID-19 positive cases among Vet Practitioners (%)	Total no. of Vet Practitioners (*n* = 112)	COVID-19 positive cases among canine (%)	Total number of dogs (*n* = 154)
Clinic 1	2 (16.7)	12	0 (0.0)	18
Clinic 2	0 (0.0)	8	1 (7.1)	14
Clinic 3	16 (100.0)	16	2 (5.0)	40
Clinic 4	9 (40.9)	22	0 (0.0)	22
Clinic 5	0 (0.0)	23	0 (0.0)	8
Clinic 6	0 (0.0)	4	0 (0.0)	12
Clinic 7	2 (22.2)	9	0 (0.0)	10
Clinic 8	0 (0.0)	4	0 (0.0)	20
Clinic 9	0 (0.0)	14	0 (0.0)	10

In the bivariate analysis (table 4), residing in Ikeja (OR: 4.12 95%CI: 1.52-11.2), exposure to positive dog (OR: 11.2 95%CI:3.67-37.18), staying in urban settlement (OR: 5.72 95%CI: 1.60-20.44), being a veterinary doctor (OR: 3.64 95%CI: 1.40-9.46), having at least tertiary education (OR: 3.74 95%CI: 1.04-13.49), and using disinfecting after attending to dog (protective) (OR: 0.15 95%CI: 0.03-0.89) were associated with being a COVID-19 case among the veterinary practitioners.

**Table 4:** Factors associated with COVID-19 infection among veterinary practitioners in Lagos (*n* = 112)

**Variable**	**Positive (%) *n* = 29**	**Negative (%) *n* = 83**	**OR (95% CI)**	***P*-value**
Gender				
Male	21 (31.8)	45 (68.2)	2.22 (0.88-5.57)	0.12
Female	8 (17.4)	38 (82.6)		
Age (years)				
< 40	18 (29.0)	44 (71.0)	1.45 (0.61-3.45)	0.52
≥ 40	11 (22.0)	39 (78.0)		
Marital status				
Married	15 (19.7)	61 (80.3)	0.38 (0.16-0.93)	0.04*
Single	14 (38.9)	22 (61.1)		
Type of clinic				
Private	16 (26.2)	45 (73.8)	1.04 (0.44-2.43)	1.00
Government	13 (25.5)	38 (74.5)		
Exposure to positive dog				
Yes	16 (66.7)	8 (33.3)	11.2 (3.67-37.18)	<0.001*
No	13 (14.8)	75 (85.2)		
Zone of resident				
Ikeja	23 (37.1)	39 (62.9)	4.12 (1.52-11.2)	0.004*
Other LGAs*	6 (12.5)	42 (87.5)		
Residential location				
Urban	26 (34.2)	50 (65.8)	5.72 (1.60-20.44)	0.003*
Semi urban	3 (8.3)	33 (91.7)		
No of H/H members				
< 5	21 (31.8)	45 (68.2)	1.63 (0.64-4.19)	0.36
≥ 5	8 (22.2)	28 (77.8)		
Cadre of staff				
Vet. doctor	22 (36.7)	38 (63.3)	3.64 (1.40-9.46)	0.01*
Others^	7 (13.7)	44 (86.3)		
Educational level				
Tertiary	26 (40.0)	58 (60.0)	3.74 (1.04-13.49)	0.05*
Below tertiary	3 (10.7)	25 (89.3)		
Years of experience				
≤ 10 years	22 (29.7)	52 (70.3)	1.87 (0.72-4.89)	0.26
> 10 years	7 (18.4)	31 (81.6)		
Working within clinic				
Yes	24 (25.0)	72 (75.0)	0.73 (0.23-2.32)	0.55
No	5 (31.3)	11 (68.7)		
Close contact with canine in clinic				
Yes	25 (26.3)	70 (73.7)	1.55 (0.41-5.89)	0.76
No	3 (18.8)	13 (81.2)		
Use alcohol based sanitizer regularly				
Yes	28	70	1.20 (0.12-12.03)	0.68
No	1	13		
Wear facemask when close to people				
Yes	24	68	1.06 (0.35-3.23)	0.58
No	5	15		
Practice social distancing				
Yes	25	63	1.98 (0.62-6.39)	0.19
No	4	20		
Goes to crowded places				
Yes	18	38	1.94 (0.82-4.61)	0.09
No	11	45		
Disinfect examination table after attending to dog				
Yes	25	81	0.15 (0.03-0.89)	0.04*
No	4	2		

In the multivariate analysis., contact with positive dog (AOR: 25.42 95%CI: 4.73-136.66), not using disinfectant after attending to dog (AOR: 12.43 95%CI: 1.39-110.99), and practicing as a veterinary doctor during the pandemic (AOR: 6.11 95%CI: 1.56-24.00), were predictors (Table 5).

**Table 5:** Predictors of Covid-19 infection among front line veterinary practitioners in Lagos State, June – August, 2020 (*n* = 112)

**Variables**	**Adjusted Odds Ratio**	**95% Confidence Interval**	***P*-value**
Cadre of staff (Vet. doctor/ Others)	6.11	(1.56-24.00)	<0.01*
Disinfecting examination table after attending to dog (No/Yes)	12.43	(1.39-110.99)	0.03*
Marital status (single/married)	0.76	(0.17-3.30)	0.71
Zone of residence (Other LGAs/ Ikeja.	0.43	(0.13-1.36)	0.15
Gender (Male/Female)	2.24	(0.60-8.38)	0.23
Exposure to positive dog (Exposed/Not exposed)	25.42	(4.73-136.66)	<0.01*
Goes to crowded places (Yes/No)	0.45	(0.13-1.59)	0.21
Educational Level (Tertiary/bellow tertiary)	1.38	(0.24-8.08)	0.72

**Fig. 2:**
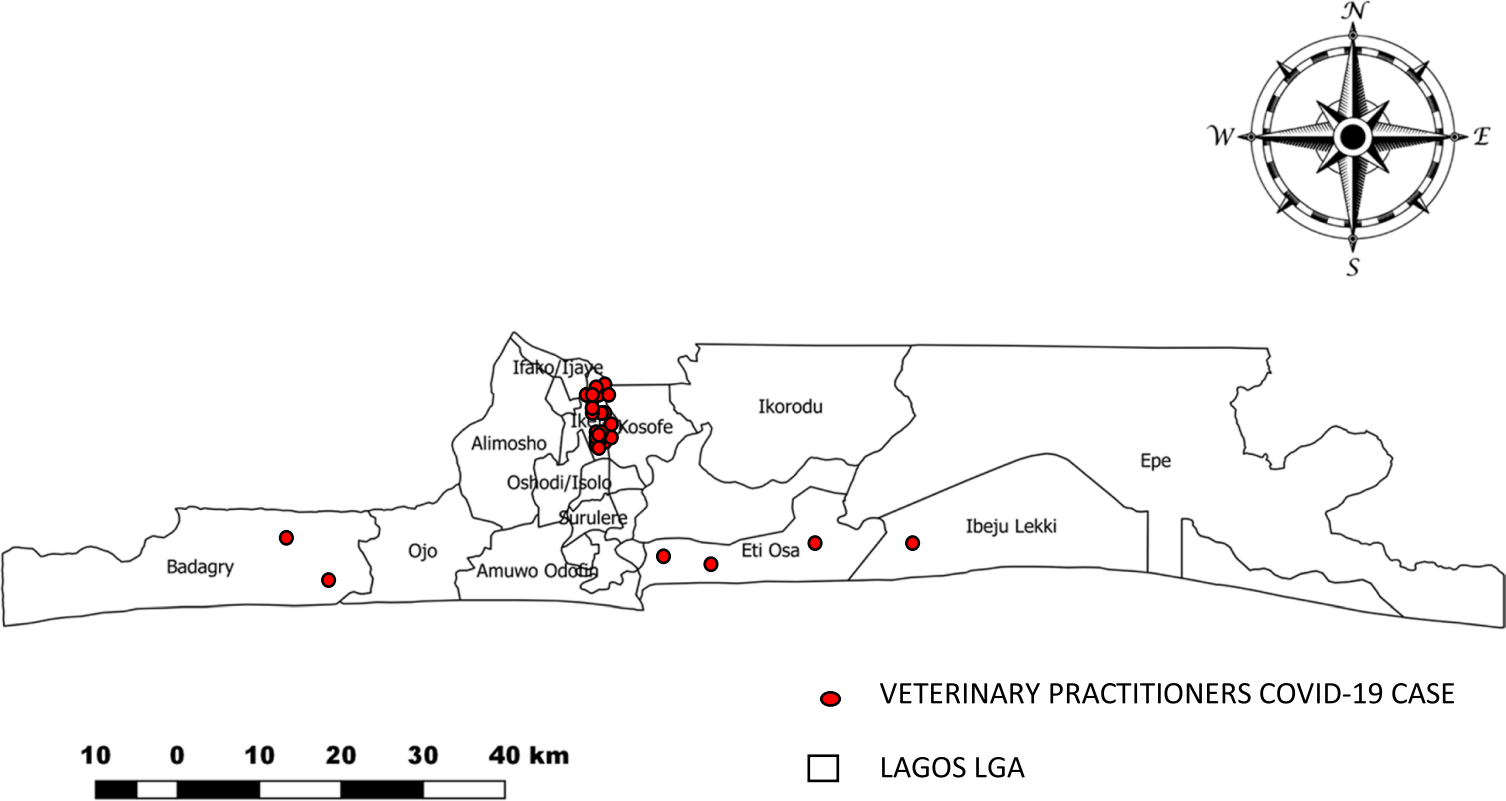
COVID-19 cases among veterinary practitioners in Lagos by LGA of residency, June to August 2020. This showed the number and distribution of positive COVID-19 cases among veterinary practitioners in Lagos State, within the period of the study. A total of 25.9% of veterinary practitioners sampled returned a positive result

**Fig. 3:**
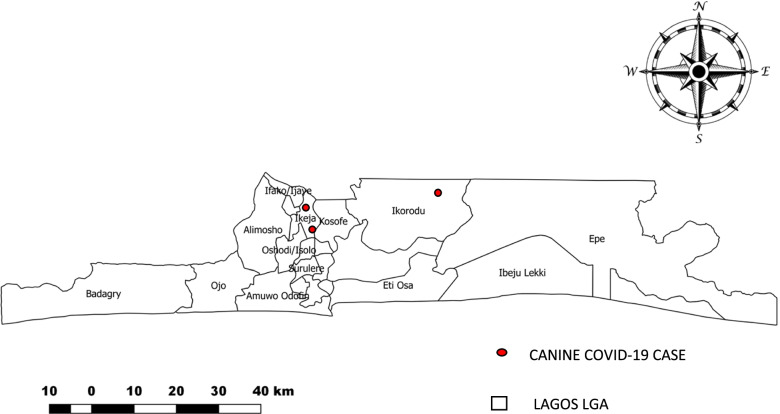
Dogs SARS-CoV-2 cases in Lagos by LGA of residency, June to August 2020. This showed the number and distribution of positive COVID-19 cases among Canine in Lagos State, within the period of the study. A total of 2.0% of canine sampled returned a positive result

A total of 23 COVID-19 positive veterinary practitioners sampled resided in Ikeja LGA, while the remaining are scattered in three other LGA in Lagos. (Fig. 2). A total of 2 SARS-CoV-2 positive canine among those sampled resided in Ikeja LGA with Ikorodu LGA recording 1 case. (Fig. 3)

## Discussion

In this study, majority of veterinary practitioners and few of the dogs attended to during the COVID-19 lockdown in Lagos were exposed to the SARS-CoV-2 virus. More so, factors such as having close contact with SARS-CoV-2 positive dogs, being a veterinary doctor attending to dog patients in clinics during the Lagos lockdown period, and not disinfecting the examination table after attending to dogs in the clinic were predictors of being a COVID-19 case among veterinary practitioners. Such knowledge can help to contain the pandemic among this group of essential service providers post lockdown, through adopting the right approach to precautionary measures.[8]

A total of 29(25.9%) of the veterinary practitioner in our study returned a positive result, among which 72.5% of cases were male with the majority of the cases being among the younger veterinary practitioners in the private sector and those staying in the urban settlement of Lagos State. This is in tandem with a 2021 gender based study of COVID-19 incidence in Nigeria, [20] which showed that more cases of the infection are seen among the male gender in urban locations than female. In our study, married practitioners were more likely to return a positive COVID-19 result, this is because they have higher possibility to be exposed to other members of their family each day than single practitioners who are likely to be living alone, although this finding was not significant at multivariate level.

Ikeja LGA was one of the epi-center of the COVID-19 infection in Lagos State, coming only behind Eti-Osa and Ibeju-Lekki LGAs. [5] Positive veterinary practitioners in Lagos came majorly from Ikeja with only 3 cases coming from Eti-Osa, 2 from Badagry and only one from Ibeju-Lekki LGAs. This distribution is related to that among the general population [5] suggesting a relationship with the community transmission of the COVID-19 infection. The distribution of infected dogs also had a relationship with the distribution of positive respondents, with more cases found among dogs that resides in Ikeja LGA.

Only 3(2%) of dogs tested for SARS-CoV-2 returned a positive result. This could be because our study was carried out quite early during the pandemic in Lagos, Nigeria. Community transmission of the infection was only confirmed in Lagos by June 2020,[21] just about the same time we commenced our study, hence more cases among animals at this stage of the pandemic might not have occurred.

Being a veterinary doctor working in Lagos both in public and private clinics during the lockdown was a risk factor for returning a positive COVID-19 result, this is because they are more at risk of close contact with their clients and patients and according to a recent national survey of similar population, [8] veterinary doctors in Nigeria were shown to have a lower practice score for carrying out COVID-19 prevention practices during the pandemic, hence their higher likelihood to return a positive result.

In our study, we found that exposure of a veterinary practitioner to a positive dog was a risk factor to their returning a positive COVID-19 result, this finding is similar to that found in a study conducted in 2020 among dogs and cats belonging to COVID-19 positive households in Italy, [22] where being exposed to a COVID-19 positive household and verse-versa was significantly associated with returning a positive result. This is a significant finding indicating the possibility of a zooanthroponotic or anthropozoonotic potential of the SARS-CoV-2 virus thereby creating a solid basis for a similar study that will make use of genomic sequencing to establish temporality of infection.

Adherence to IPC etiquettes, during the pandemic, has proven to be a major way to contain the disease at all levels in the society, non-pharmaceutical prevention measures like social distancing, wearing of face masks in public places, regular washing of hands, and use of hand sanitizers has been recommended by the US CDC and other public health organization worldwide. In our study, we found out that veterinary practitioners that disinfect their examination table after attending to dogs were protected from the COVID-19 infection as against those that did not adhere to this practice.

We however, recognize that our study has some limitations. Being the foremost survey on SARS-CoV-2 infection among dogs in Lagos, we had the challenge of the right test kits to use for diagnosis among the animal subjects, as RT-PCR, which was the gold standard for COVID-19 diagnosis was unavailable for animal subjects at the time our study was conducted. However, we were able to determine possible exposure of dogs in the study area to SARS-CoV-2 virus using serological test. Further study is required when SARS-CoV-2 diagnostic test kits for animals especially dogs are readily available for a better diagnostic sensitivity and specificity. We were also limited in our ability to determine the temporal relationship that exist between dogs and human cases, due to the cross-sectional study design adopted, but an association was established which was a significant addition to existing knowledge as at the time the study was conducted.

## Conclusion

The prevalence of COVID-19 infection is relatively high among veterinary practitioners and low among dogs in Lagos, Nigeria from June to August 2020. Predictors of COVID-19 case among veterinary practitioners included contact with a SARS-CoV-2 infected dog, being a veterinary doctor working during the lockdown period, and not disinfecting examination table after attending to dogs in the clinic. We recommend that veterinary clinics should routinely screen dogs with clinical signs consistent with SARS-CoV-2 infection being admitted into their facility and ensure the judicious use of disinfectants after attending to dogs. Infection prevention and control measures should also be strictly adhered to by veterinary doctors when attending to dogs and clients in the clinic.

## Supplementary Information

**Additional file 1: **A Microsoft excel xlsx file format. Social demographic distribution of veterinary practitioner respondents during our study from June to August 2020. This file shows the data from which figure 2 was derived and also showed the selected LGA in which Figure 1 highlights. The COVID-19 status response and the LGA of residents of respondents are indicates on column C and G of the spreadsheet respectively.

**Additional file 2: **A Microsoft excel xlsx file format. Data used for multivariate analysis of risk factors responsible for the occurrence of COVID-19 infection among veterinary practitioners in Lagos June to August 2020. This is the file from which the multivariate analysis of significant risk factors from the bivariate analysis of table 3 was carried out, the result of this analysis gave rise to the result in table 5.

## Data Availability

All data generated or analyzed during this study are included in this published article [and its supplementary information files].

## Abbreviations

LGA: Local government area
CFR: Case fatality rate
WHO: World health organization
OR: Odd ratio
IPC: Infection prevention and control
FCT: Federal capital territory
NGO: Non-governmental organization
UN: United nations
DVM: Doctor of Veterinary Medicine
NCDC: Nigerian Center for Disease Control
PCR: Polymerase Chain Reaction
IgG/IgM: Immunoglobulin G/ Immunoglobulin M
